# Six year disease free survival after liver transplantation in a patient with T3 gallbladder carcinoma: case presentation and review of the literature

**DOI:** 10.1186/1477-7819-4-45

**Published:** 2006-07-14

**Authors:** Jorge Ortiz, David Reich, Hoon Bae Joon, Oscar Martinez, Cosme Manzarbeitia

**Affiliations:** 1 Texas Transplant Institute, 8201 Ewing Halsell #280, SanAntonio, Texas 78229, USA; 2 Albert Einstein Medical Center, 5501 Old York Road, Center forLiver Diseases, Philadelphia, PA 19141, USA; 3 University of Kentucky, 800 Rose Street, Division ofTransplant, Lexington, KY 40536, USA

## Abstract

**Background:**

The incidence of gallbladder carcinoma in cirrhotics is unknown. Known risk factors are primary sclerosing cholangitis and polypoid masses.

**Case presentation:**

A sixty year old with primary sclerosing cholangitis, cirrhosis, and gallbladder polyps underwent liver transplantation. A polypoid lesion measuring 1.5 × 0.5 cm was found on the fundus of the gallbladder. Histological examination revealed moderately differentiated adenocarcinoma with full thickness penetration of the gallbladder encroaching liver parenchyma. Angiolymphatic invasion was noted. The lymph nodes, the cystic duct and the common duct were free of tumor (T3N0M0). Extensive evaluation did not demonstrate metastasis. No chemotherapy was given. He is currently six years post procedure and free of disease.

**Conclusion:**

"Incidentally" discovered stage IIA gallbladder carcinoma may not negatively affect long term survival after liver transplantation.

## Background

Between 6000 and 7000 cases of gallbladder carcinoma are diagnosed yearly in the United States. Advanced gallbladder carcinoma (GBC) usually portends an extremely grave prognosis [1]. Recent reports in the literature have demonstrated improved results after aggressive surgical resection for stage II–IV lesions [2]. Although there are sporadic reports of incidental gallbladder carcinoma found after liver transplantation, no consensus exists on the proper treatment for those patients found to have gallbladder carcinoma with cirrhosis.

Major risk factors for the development of GBC include polypoid lesions and sclerosing cholangitis (PSC). Larger polyps, especially in the setting of PSC (with or without cirrhosis) have a greater than 50% chance of harboring malignancy [3]. Primary sclerosing cholangitis is a chronic progressive disorder of unknown etiology that is characterized by inflammation, fibrosis, and stricturing of medium size and large ducts in the intrahepatic and extraheptic biliary tree [4]. The only definitive therapy is liver transplantation. There are no biochemical or clinical risk factors for the development of hepatobiliary cancer in patients with PSC [5].

Ultrasound and CT scan are usually relied upon to diagnose polypoid lesions and other abnormalities of the gallbladder. However, due to chronic cholecystitis, wall thickening and gallstones, the lesions are frequently missed [6].

We present the case of a gentleman with primary sclerosing cholangitis and a polypoid lesion of the gallbladder who underwent liver transplantation. Pathology revealed a T3N0M0 (AJCC 2002) gallbladder carcinoma. He is currently six years post procedure without any signs of recurrent disease. Although there are case presentations of patients with incidental and early (T1 and T2) lesions demonstrating excellent medium term survival, this is the first report of a patient with a T3 lesion attaining six year disease free survival after liver transplantation.

## Case presentation

A sixty year old male diagnosed with primary sclerosis cholangitis and cirrhosis caused by primary sclerosing cholangitis after an ERCP and liver biopsy in June of 1999. A computerized tomographic scan (CT) revealed a cirrhotic liver, enlarged spleen, coronary vein and gallbladder wall varices and a polypoid mass of the gallbladder confirmed on follow up ultrasonography. Due to the patient's debilitated state and risk for life threatening decompensation, the decision was made not to remove the gallbladder.

**Figure 1:**
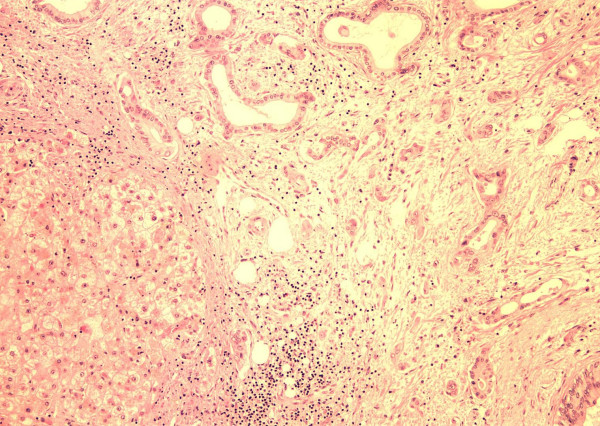
Infitrating gallbladder carcinomia (H & E 40 ×).

**Figure 2:**
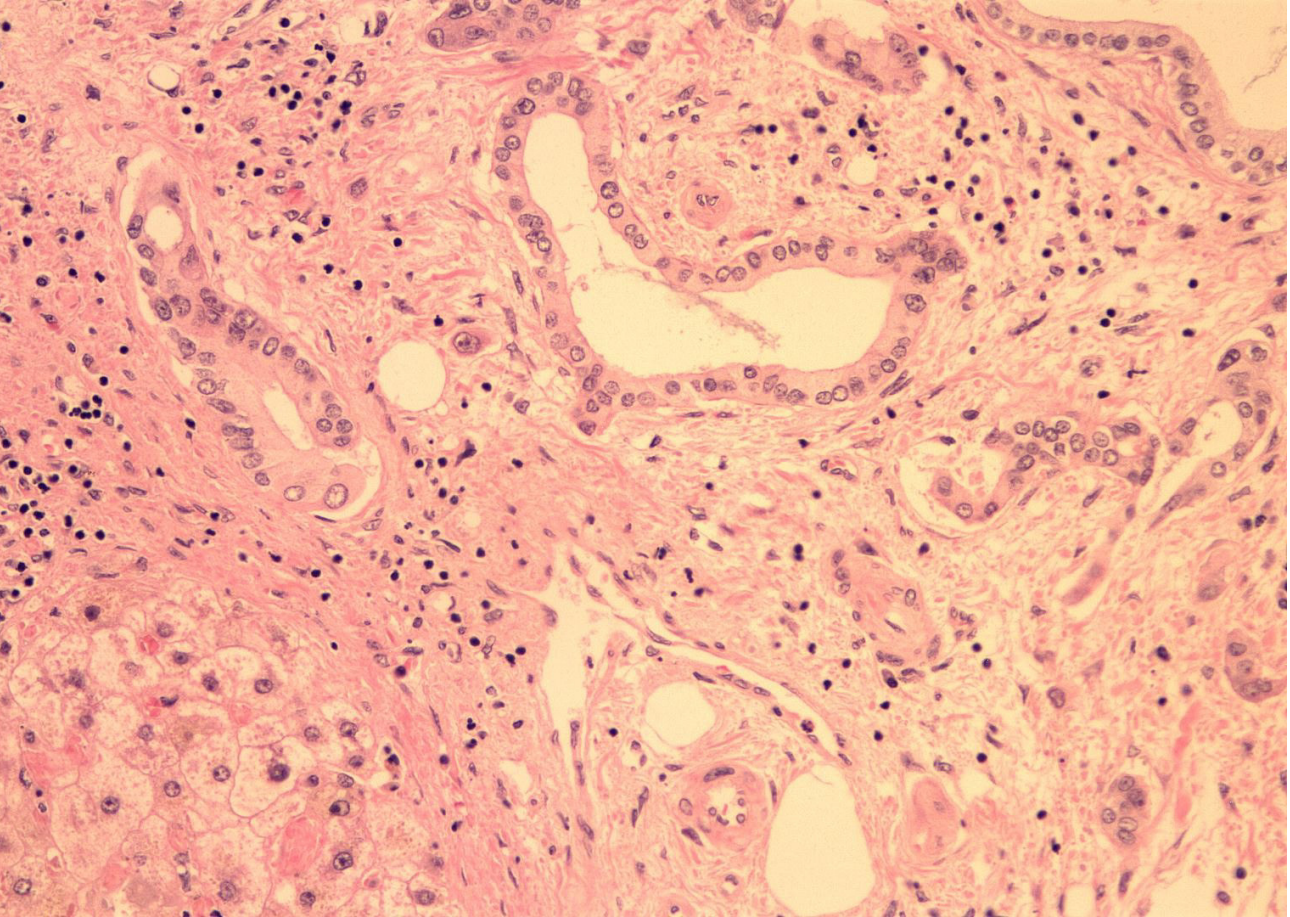
Angiolymphatic Invasion (H & E 400 ×).

**Figure 3:**
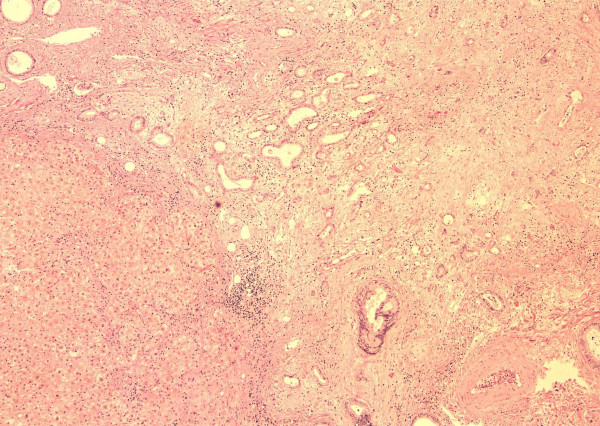
Encroached Liver Parenehyma (H & E 20 ×).

He was listed for liver transplantation as status 2A. On November 16th, 1999 an ABO compatible size matched organ was allocated to him. The transplant was performed with the conventional technique with venovenous bypass. In the explanted liver, a polypoid mass, which measured 1.5 × 0.5 cm, was found on the fundus of the gallbladder. Histological examination revealed moderately differentiated adenocarcinoma with full thickness penetration of the gallbladder encroaching (within millimeters) liver parenchyma. Angiolymphatic invasion was confirmed. (Figures 1, 2, 3) Examination of the hepatic hilar lymph nodes, common duct, cystic duct and the remainder of the liver did not show any metastasis (T3N0M0, Stage IIA; AJCC 2002).

Extensive metastatic evaluation was also negative. The patient recovered uneventfully with triple immunosuppression (Tacrolimus, mycophenalate, and prednisone) and received no chemotherapy.

Patient was being followed closely in the liver transplant clinic and had normal liver function tests until his demise in June of 2006. A CT scan performed June 2006 was negative for any signs of gallbladder carcinoma.

## Discussion

Primary sclerosing cholangitis (PSC) is a chronic cholestatic disease that progresses to end – stage liver disease. The only definitive form of therapy is liver transplantation (LT). LT results in patient and graft survivals approaching that of other indications [7]. Patients with sclerosing cholangitis are at increased risk for cholangiocarcinoma (CC)[8]. CC develops in 10–15% of PSC patients followed for five years and up to 30% of patients followed for more than 10 years [9]. Other malignancies associated with PSC include colon, hepatocellular, fibrolamellar, and gallbladder carcinoma [10].

The exact risk of gallbladder carcinoma with sclerosing cholangitis is unknown. In a study of 25 PSC patients with a biliary malignancy, eight of the tumors were located in the gallbladder [11]. A recent Canadian report documented the presence of an occult GBC in one out of 111 patients transplanted for primary sclerosing cholangitis. The stage of that cancer is unknown [12]. The Johns Hopkins group documented one case of GBC out of 146 patients (not all transplant recipients) with primary sclerosing cholangitis treated over a 14 year time span [9]. In 127 transplanted patients with PSC at UCLA no gallbladder carcinomas were reported [7]. In 51 patients transplanted by the Lahey group over a 17.5 year period, one was found to have an "incidental" carcinoma of the gallbladder [13]. The same authors published case presentations nine years prior of two gallbladder carcinomas found incidentally after recipient hepatectomy. One patient with PSC (presumably the same patient as in the aforementioned report) had a papillary growth with a T2NoMo gallbladder carcinoma. The other patient was transplanted for primary biliary cirrhosis and was harboring a T1NoMo GBC. Both patients survived long term (2.5 years) without any further interventions [14]. In 1998, a case presentation was published of a woman with PSC and cirrhosis who underwent a cholecystectomy for a new 11 × 13 mm polyp and was found to have a GBC infiltrating all layers (a T2 lesion) and invading local lymphatic vessels. One month later an orthotopic liver transplant was performed and she was free of disease at 24 months [15].

Our patient's GBC was also discovered incidentally. However, he appears to have more extensive spread than previously documented in the literature.

GBC is the most common malignant tumor of the biliary tract [16]. The incidence in the United States is about 2.5 per 100,000 and is associated with approximately 2500 deaths per year in the U.S. [17]. In most series the five year survival is less than 10%. In 15–20% of patients it is discovered incidentally after routine cholecystectomy. Gallbladder carcinoma is found pathologically after 1–2% of all cholecystectomies. Gallstones, choledochal cysts, calcified gallbladders, anomolous pancreaticobiliary duct junctions, obesity, estrogens, and smoking [18] have been linked with the development of this disease.

The presence of polyps is also a predisposing factor for GBC [19]. The prevalence of polypoid lesions of the gallbladder is up to14% [5]. Most are benign, however those which are greater than 10 mm in diameter have the greatest malignant potential [12]. Endoscopic ultrasound may be superior to standard ultrasound and CT scan for diagnosis [6]. According to one report, gallbladder polyps in patients with primary sclerosing cholangitis are frequently (57% of polyps) malignant [3]. The mean size of the lesions in this study was 21 mm. In our patient, the polyp was appreciated on both ultrasound and CAT scan and was greater than 10 mm in its greatest dimension. However, since the risk of decompensation after cholecystectomy was significant, the decision was made not to remove the lesion prior to transplantation.

Most carcinomas of the gallbladder are adenocarcinomas. The most common mode of spread is direct invasion into liver segments IV and V and into the duodenum, colon, anterior abdominal wall and common hepatic ducts. Metastasis may involve the lymph nodes posterior to the pancreas and the portal vein. Fortunately, our patient did not demonstrate such spread.

Gallbladder carcinoma is usually diagnosed on ultrasound [6]. This modality however is notoriously inaccurate [20]. Patients with PSC are reported to have large, thickened and chronically inflamed gallbladders [21], perhaps exacerbating the inability to preoperatively diagnose these lesions. The extent of carcinoma spread is evaluated with CT scan.

In our case, the lesion was suspected but obfuscated by the gallbladder varices and chronic cholecystitis frequently seen in this scenario. The AJCC 2002 TNM classification is used in order to stage gallbladder carcinoma.

T1 lesions are best treated with simple cholecystectomy. Five-year survival approaches 100%.

Patients with T2 lesions should have a radical cholecystectomy. This will increase five year survival from 10–40% (seen with simple cholecystectomy) to 80–90% [2]. T3 or T4 lesions may require a formal segmentectomy for cure. Common duct excision and hepaticojejunostomy may be necessary for lesions involving the cytic duct common duct confluence. Five-year survivals ranging as high as 60% for T3 lesions and 25% for T4 lesions have been documented after radical resection. Some researchers believe that radical surgery should only be attempted if there is no lymph node involvement and if complete extirpation of the lesion is possible [2]. Others believe that excellent results (80% five year survival) can be obtained despite positive lymph nodes [22]. Patients at the Mayo clinic who underwent a radical cholecystectomy had a significantly longer median survival (24 months) than patients who had a simple cholecystectomy (6 months) or noncurative treatment (4 months). This was true for all stages except stage 1[22]. Survival rates without surgery are dismal [20,24].

In our case of T3 gallbladder carcinoma with PSC cirrhosis, liver transplantation may have cured the patient.

Chemotherapy has not been widely studied for the treatment of gallbladder carcinoma. Gemcitabine has shown promise in the treatment of biliary tract cancers and may have a role in treating GBC. 5-FU and cisplatin are the standard chemotherapeutic agents used [25]. At this point, absent the demonstration of therapeutic efficacy, no specific chemotherapeutic regimen can be recommended. In the setting of liver transplantation, chemotherapy is problematic because of toxicity and lack of proven efficacy. For this reason, no chemotherapy was administered to our patient.

Given the dismal results for known cholangiocarcinoma with liver transplantation [7] and the overall poor survival for extensive gallbladder carcinoma in the general population [16], liver transplantation is not recommended for known GBC.

## Conclusion

We present the case of a sixty year old gentleman with primary sclerosing cholangitis and a polypoid lesion of the gallbladder. He successfully underwent liver transplantation and was found to be harboring a T3N0M0 gallbladder carcinoma. He is currently six years post procedure and has no evidence of disease. Others have reported excellent medium term survival for incidental (T1 and T2) lesions discovered on explant. This case demonstrates that incidental gallbladder carcinoma (like incidental cholangiocarcinoma) may not negatively affect long term survival after liver transplantation. However, further studies are needed in order to evaluate the usefulness of liver transplantation in the setting of advanced gallbladder carcinoma discovered before transplantation.

## Declaration of competing interests

The author(s) declare that they have no competing interests.

## Authors' contributions

**JO **Conceived of the idea and drafted the manuscript. **DR **and **CM **performed surgical procedure and did patient follow up. **HJ **and **OM **did the research and data collection.

All authors read and approved the final manuscript.
